# Effect of a hydrogel spacer on the intrafractional prostate motion during CyberKnife treatment for prostate cancer

**DOI:** 10.1002/acm2.13005

**Published:** 2020-08-10

**Authors:** Toshihiro Suzuki, Masahide Saito, Hiroshi Onishi, Zennosuke Mochizuki, Koji Mochizuki, Kenichiro Satani, Naoki Sano, Shinichi Aoki, Kan Marino, Takafumi Komiyama, Hiroshi Takahashi

**Affiliations:** ^1^ Kasugai CyberKnife Rehabilitation Hospital Yamanashi Japan; ^2^ Department of Radiology University of Yamanashi Yamanashi Japan

**Keywords:** CyberKnife, fiducial marker, hydrogel spacer, prostate cancer

## Abstract

The purpose of this study was to evaluate the effect of a hydrogel spacer on intrafractional prostate motion during CyberKnife treatment. The retrospective study enrolled 24 patients (with the hydrogel spacer = 12, without the hydrogel spacer = 12) with two fiducial markers. Regarding intrafractional prostate motion, the offset values (mm) of three axes (X‐axis; superior [+] to inferior [−], Y‐axis; right [+] to left [−], Z‐axis; posterior [+] to anterior [−]) obtained from fiducial markers position between a digitally reconstructed radiographs images and live images in the Target Locating System were used, and extracted from generated log files. The mean values of the offset and each standard deviation were calculated for each patient, and both the groups were compared. For all the patients, a total of 2204 offset values and timestamps (without the hydrogel spacer group: 1065, with the hydrogel spacer group: 1139) were recorded for the X‐, Y‐, and Z‐axes, respectively. The offset values (mean ± standard deviation) for the X‐, Y‐, and Z‐axes were −0.04 ± 0.92 mm, −0.03 ± 0.97 mm (*P* = 0.66), 0.02 ± 0.51, −0.02 ± 0.49 mm (*P* = 0.50), and 0.56 ± 0.97 mm, 0.34 ± 1.07 mm (*P* = 0.14), in patients inserted without or with the hydrogel spacer, respectively. There was no effect of a hydrogel spacer on the intrafractional prostate motion in the three axes during CyberKnife treatment for prostate cancer.

## 1 INTRODUCTION

External beam radiotherapy (EBRT) for the treatment of localized prostate cancer involves the use of several techniques such as three‐dimensional conformal radiotherapy (3DCRT), intensity‐modulated radiotherapy (IMRT), volumetric modulated arc radiotherapy (VMAT), proton therapy and stereotactic body radiotherapy (SBRT).^1^, ^2^, ^3^, ^4^, ^5^ The use of a higher dose with high‐precision EBRT techniques results in better rate of cancer control.^6^ However, high doses might also be delivered to the surrounding normal tissue, thereby possibly affecting the patient quality of life.^7^, ^8^ Accordingly, rectal dose reduction is important while considering the late toxicities after radiotherapy for prostate cancer.^9^, ^10^ Recently, a hydrogel spacer (SpaceOAR^TM^ System, Augmenix Inc., Waltham, MA) was introduced, and it helped reducing rectal toxicities via the insertion of an absorbable hydrogel between the prostate and rectum.^11^, ^12^ The use of a hydrogel spacer stably reduced the rectal dose in all EBRT modalities.^13^ Moreover, for high‐precision EBRT, it is important to control inter‐ and intrafractional prostate motion. Among the several radiotherapy modalities, CyberKnife (Accuray, Sunnyvale, CA) can be used to acquire 2D images per 5–150 s by using 2‐kV x‐ray devices (target locating system [TLS]), resulting in high‐precision image‐guided radiotherapy.^14^ Prostate SBRT using CyberKnife showed fewer grade 2 or worse genitourinary toxicities than other SBRT delivery methods.^15^ However, larger intrafractional motion was observed in CyberKnife treatment than in conventional Linac treatment, because the irradiation time for CyberKnife treatment was likely to be long. Accordingly, we hypothesized that the use of a hydrogel spacer would help in fixing the position of the prostate, thereby reducing its movement. To the best of our knowledge, no previous studies have compared prostate motion between patients with and without a hydrogel spacer during CyberKnife treatment. Therefore, the current study aimed to evaluate the effect of placing a hydrogel spacer on intrafractional prostate motion during CyberKnife treatment.

## 2 MATERIALS AND METHODS

### 2.A Patient data

This retrospective study was reviewed and approved by the Institutional Review Board of our institution. A total of 24 patients who underwent CyberKnife treatment for prostate cancer between March 2017 and May 2020 in our institution were enrolled in the study. Table 1 lists the patient characteristics. All the patients were implanted with one fiducial marker each in the left and right lobes of the prostate. The fiducial marker used was either the ball‐shaped Gold Anchor (0.28 mm × 10.0 mm; Naslund Medical AB, Huddinge, Sweden) or the straight‐shaped VISICOIL (0.5 mm × 5.0 mm; RadioMed Corporation, Bartlett, TN). In addition, radiation oncologists inserted the hydrogel spacer between the prostate and rectum under transrectal ultrasound guidance in 12 patients.

**Table 1:** Patients and tumor characteristics (n = 24). *P*‐value is calculated by Wilcoxon test or Fisher's exact test as appropriate.

Characteristics	Hydrogel spacer (−)	Hydrogel spacer (+)	*P*‐value
Patients (n)	12	12	
Age (years)			
Median (range)	72 (66–85)	70 (65–80)	0.06
Volume (cc) (Median (range))	33.5 (21–113)	41.9 (11.8–102.2)	0.71
Gleason score (≤6/7/8≤)	3/4/5	4/6/2	0.58
TNM(T1/T2/T3)	4/7/1	1/9/2	0.46
Treatment time (min) Mean (range)	50.3 (38–64)	54.8 (42–71)	0.18
Patient with no manual alignment	7/12	6/12	1.00
Patient with manual alignment	5/12	6/12	1.00
(Number of manual alignment/total time stamp)	Patient 2: (2/97) Patient 3: (2/94) Patient 5: (2/77) Patient 7: (1/95) Patient 10: (4/101)	Patient 13: (4/93) Patient 14: (1/91) Patient 17: (5/83) Patient 19: (2/95) Patient 21: (4/106) Patient 22: (5/110)	

### 2.B Treatment planning

All computed tomography (CT) images were acquired using Optima CT660 (GE MedicalSystems, Milwaukee, WI) with following settings: 120 kV, 400 mA, 1.25‐mm slice thickness, 500‐mm field of view, and 512 × 512 pixels. Treatment planning was performed using CT (1.25‐mm slice) and magnetic resonance imaging (T2, T2*) with the CyberKnife MultiPlan TPS (Accuray, Sunnyvale, CA). The planning target volume (PTV) margin was prepared by adding 3 mm in the posterior direction and 5 mm in other directions to the clinical target volume; a total dose of 36.25 Gy was delivered to the PTV in 5 fractions. 104–317 noncoplanar beams were used per fraction. Then, the treatment time was optimized to obtain the acceptable time. All patients were required to present with a full bladder and empty rectum at the time of CT simulation and each treatment fraction.

### 2.C Fiducial tracking

During the CyberKnife treatment of prostate cancer, the fiducial tracking system monitors fiducial markers near the treatment site. This system calculates the offset values and corrects it when the marker array differs between live images and digitally reconstructed radiographs (DRR) images in the TLS.^14^ For the fiducial tracking system, the offset values (mm) of the three axes (X‐axis; superior [+] to inferior [−], Y‐axis; right [+] to left [−], and Z‐axis; posterior [+] to anterior [−]) can be obtained by using one or two fiducial markers, although the correction for the other axis (pitch, roll, and yaw [degrees]) needs a minimum of three fiducial markers. These values are recorded in the system log files.^16^, ^17^, ^18^ Before starting the treatment, the acceptable offset values for all the three axes are setup within ±1.0 mm by using corresponding couch shift. Then, a threshold of ±3 mm is normally used as an acceptable value during treatment in our institution (if the offset value of any axis was more than 3.0 mm during treatment, the treatment couch was manually corrected). In the current study, the irradiation interval for TLS was set as 120 s (17% of all fractions), 140 s (15% of all fractions), or 150 s (68% of all fractions).

### 2.D Procedure

In the current study, prostate motion was defined using the offset values in each direction. Data in the log files of the 24 patients with prostate cancer treated with fiducial tracking were analyzed. The mean values and standard deviation of the offset value for each axis were obtained both with and without the hydrogel spacer. The t‐test was used to compare values between the groups. All data analyses were performed using the R statistical package.

## 3 RESULTS

**Fig. 1:**
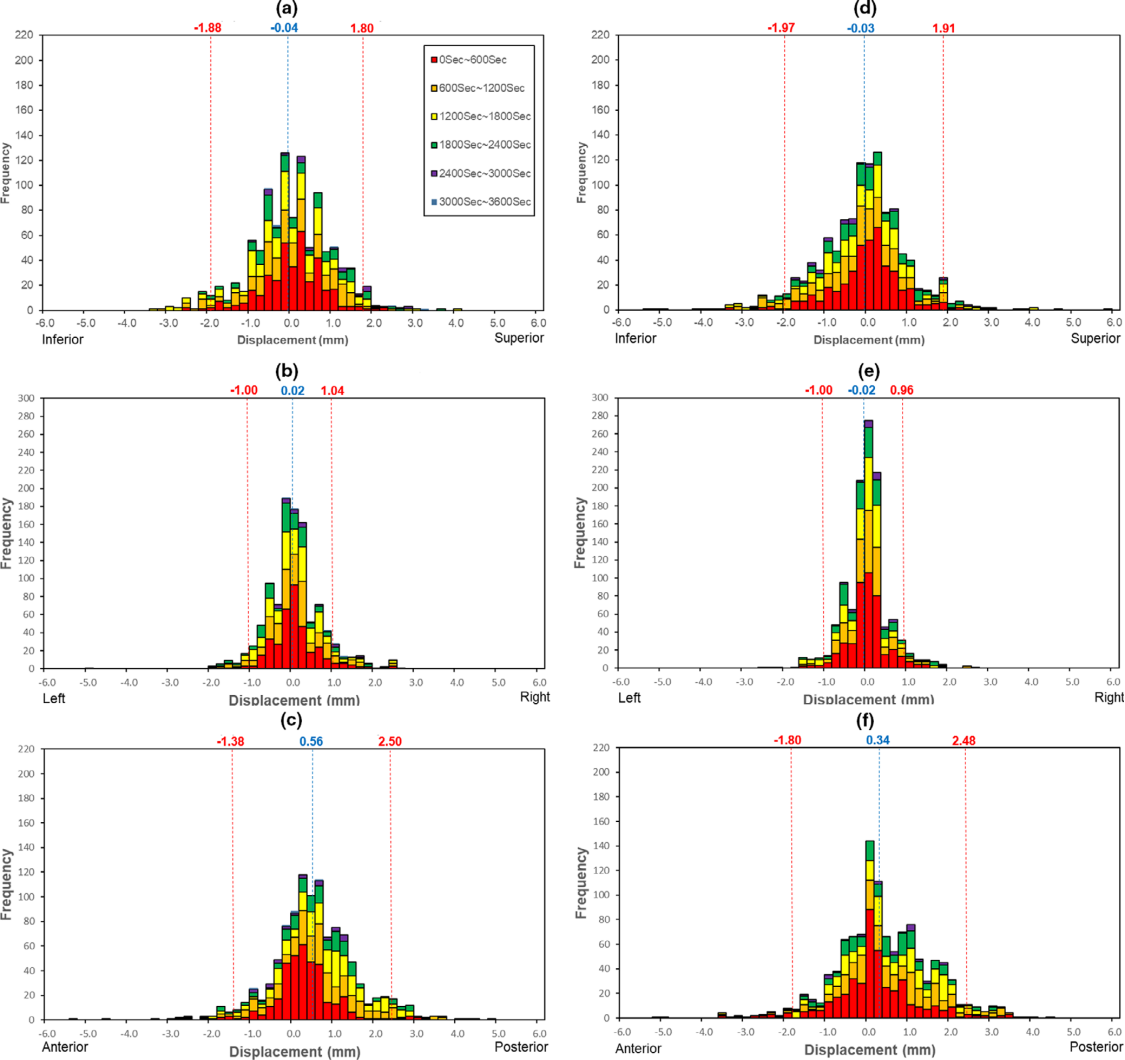
A histogram showing prostate motion in the treatment period as a function of intrafractional time and frequency of offset values. The displacements of the x‐axis (a and d), y‐axis (b and d), and z‐axis (c and e) are shown. The images in the left column (a, b, c) show patients treated without a hydrogel spacer, and those in the right column (d, e, f) show the patients treated with a hydrogel spacer. The histogram is divided into specific time segments per 600 s, indicated using different colors. Each histogram includes the mean displacement (blue) and 95% confidential interval (red).

For the 24 patients, a total of 2204 offset values and timestamps (1065 for the patients in the group without the hydrogel spacer, and 1139 for the patients in the group with the hydrogel spacer, respectively) were recorded for the X‐, Y‐, and Z‐axes. The treatment time was 38–71 min. During the treatment, manual alignment to correct the prostate position within the threshold value in patients treated without a hydrogel spacer was performed in 2/97, 2/94, 2/77, 1/95, and 4/101 (number of manual alignment/total time stamp) for patients 2, 3, 5, 7, and 10, respectively. In the same manner, manual alignment in patients treated with a hydrogel spacer was performed in 4/93, 1/91, 5/83, 2/95, 4/106, and 5/110 (number of manual alignment/total time stamp) for patients 13, 14, 17, 19, 21, and 22, respectively. (Table 1). Table 2 lists the offset values for all the directions. The offset values (mean ± standard deviation) for the X‐axis were −0.04 ± 0.92 mm and −0.03 ± 0.97 mm (*P* = 0.66) for the patients without and those with the hydrogel spacer, respectively. Similarly, the corresponding offset values for the Y‐axis were 0.02 ± 0.51 mm and −0.02 ± 0.49 mm (*P* = 0.50) while those for the Z‐axis were 0.56 ± 0.97 mm and 0.34 ± 1.07 mm (*P* = 0.14). Both groups had larger mean value for the Z‐axis than for the other axes. Figure 1 shows the histogram of the prostate motion in the treatment period as a function of intrafractional time and frequency of offset values. The spread of the spatial distribution for all axes in both groups at 0–600 s was narrow. In particular, the spread of the spatial distribution of the whole plot was narrow for the Y‐axis. In contrast, the plots of the Z‐axis were distributed to the posterior side with the lapse of time.

**Table 2:** The offset values (mean ± standard deviation) for each axis. Both groups had larger mean value for the Z‐axis than for the other axis.

	Mean ± S.D. (mm)		
	Hydrogel spacer (−)	Hydrogel spacer (+)	*P*‐value
X‐axis (mm)	−0.04 ± 0.92	−0.03 ± 0.97	0.66
Y‐axis (mm)	0.02 ± 0.51	−0.02 ± 0.49	0.50
Z‐axis (mm)	0.56 ± 0.97	0.34 ± 1.07	0.14

## 4 DISCUSSION

**Fig. 2:**
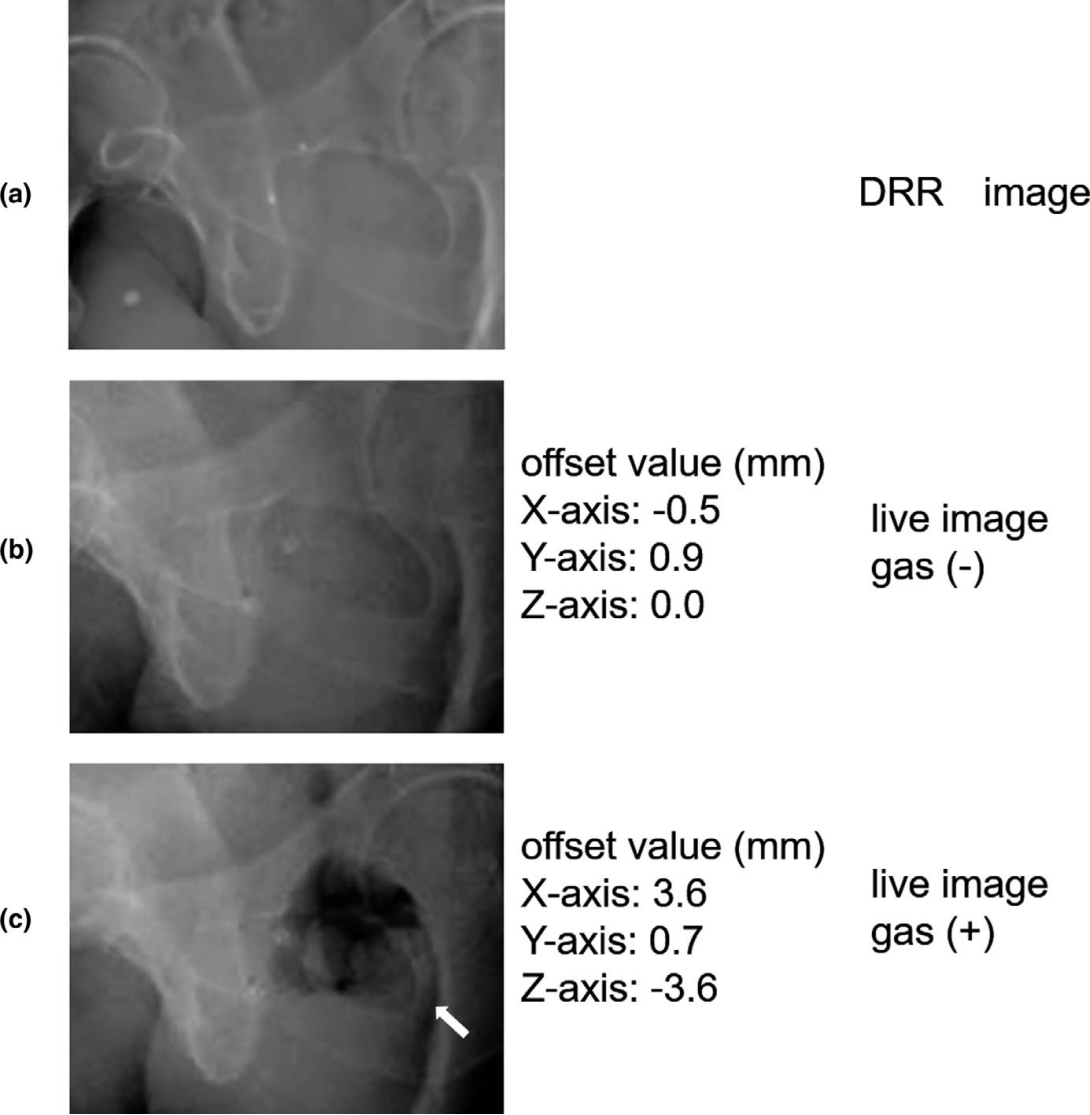
Typical images during treatment of a patient with a lot of rectal gas: (a): DRR image, (b): live image of a patient without gas, (c): live image of a patient with gas (arrow).

We hypothesized that using a hydrogel spacer would fix the position of the prostate and reduce its movement during the CyberKnife treatment. However, our results indicated that there were no significant differences between the groups, although the mean values were higher for the Z‐axis than for the other axes. Several studies have investigated the prostate motion in the anterior–posterior (AP) dimension during CyberKnife treatment; however, these studies have not shown consistent results.^19^, ^20^ In contrast, a review article on prostate motion in general radiotherapy showed the tendency for the large motion in the AP dimension, which was consistent with our results. It was thought that bladder distension, rectal peristalsis, and anal contraction were the main factors that affected the prostate motion.^21^, ^22^ In addition, manual alignment to correct the prostate position was performed more frequently in those treated with a hydrogel spacer than in those treated without a hydrogel spacer. Figure 2 shows typical images during the treatment of a patient with a lot of rectal gas. Several previous studies have investigated the effect of using a hydrogel spacer on prostate motion throughout radiotherapy, similar to using endorectal balloons.^20^, ^23^, ^24^, ^25^, ^26^ The offset of the three translational axes was not significantly different between the bony anatomy and fiducial markers obtained via planning CT and cone beam CT performed after weekly setup, irrespective of the use of a hydrogel spacer.^24^ In contrast, the use of a hydrogel spacer reduced posterior displacement of the prostate when the distance was measured between the prostate and the anterior rectum on planning CT and CT scans obtained in the last week of radiation.^25^ Intrafraction prostate motion with the use of a hydrogel spacer was shown by Juneja et al. and Sumila et al.^20^, ^26^. Juneja et al. reported that using a hydrogel spacer did not affect the three axes, and the treatment time was short. In contrast, Sumila et al. reported that prostate motion was observed during CyberKnife treatment with a long treatment time. However, the current study is the first to evaluate intrafractional motion with and without a hydrogel spacer during CyberKnife treatment. We showed that the use of a hydrogel spacer did not affect any of the three axes of prostate motion.

The current study has some limitations. Although the use of three or more fiducial markers in the prostate is recommended, only two markers were used in the current study, as only two are reimbursed by the Japanese medical insurance system. In addition, the effects of using a hydrogel spacer could not be compared in the same patient. Finally, in our study, it would be difficult to perfume this procedure with the same frequency as that of manual alignment in the two groups. Furthermore, this study investigated prostate motion only in the three axes. Future studies should be including pitch, roll, and yaw.

## 5 CONCLUSION

We evaluated the effect of using a hydrogel spacer on intrafractional prostate motion in the three axes. No significant differences in prostate motion were observed between patients treated without or with a hydrogel spacer. Our study suggested that a hydrogel pacer could not affect on intrafractional prostate motion during CyberKnife treatment.

## CONFLICT OF INTEREST

There are no conflict of interest to declare.

## References

[1] Zelefsky MJ , Fuks Z , Happersett L , et al. Clinical experience with intensity modulated radiation therapy (IMRT) in prostate cancer. Radiother Oncol. 2000;55:241–249. 10869739 10.1016/s0167-8140(99)00100-0

[2] Chen LN , Suy S , Uhm S , et al. Stereotactic body radiation therapy (SBRT) for clinically localized prostate cancer: the Georgetown University experience. Radiat Oncol. 2013;8:58. 23497695 10.1186/1748-717X-8-58 PMC3610192

[3] Anwar M , Weinberg V , Chang AJ , Hsu I‐C , Roach M , Gottschalk A . Hypofractionated SBRT versus conventionally fractionated EBRT for prostate cancer: comparison of PSA slope and nadir. Radiat Oncol. 2014;9:42. 24484652 10.1186/1748-717X-9-42 PMC3923240

[4] King CR , Lehmann J , Adler JR , Hai J . CyberKnife radiotherapy for localized prostate cancer: rationale and technical feasibility. Technol Cancer Res Treatm. 2003;2:25–29. 10.1177/153303460300200104 12625751

[5] Slater JD , Rossi CJ Jr , Yonemoto LT , et al. Proton therapy for prostate cancer: the initial Loma Linda University experience. Int J Radiat Oncol Biol Phys. 2004;59:348–352. 15145147 10.1016/j.ijrobp.2003.10.011

[6] Michalski JM , Moughan J , Purdy J , et al. Effect of standard vs dose‐escalated radiation therapy for patients with intermediate‐risk prostate cancer: the NRG oncology RTOG 0126 randomized clinical trial. JAMA Oncol. 2018;4:e180039. 29543933 10.1001/jamaoncol.2018.0039 PMC5885160

[7] Katz AJ , Santoro M , Ashley R , Diblasio F , Witten M . Stereotactic body radiotherapy for organ‐confined prostate cancer. BMC Urol. 2010;10:1. 20122161 10.1186/1471-2490-10-1 PMC2831888

[8] Zelefsky M , Leibel S , Gaudin P , et al. Dose escalation with three‐dimensional conformal radiation therapy affects the outcome in prostate cancer. Int J Radiat Oncol Biol Phys. 1998;41:491–500. 9635694 10.1016/s0360-3016(98)00091-1

[9] Zelefsky MJ , Levin EJ , Hunt M , et al. Incidence of late rectal and urinary toxicities after three‐dimensional conformal radiotherapy and intensity‐modulated radiotherapy for localized prostate cancer. Int J Radiat Oncol Biol Phys. 2008;70:1124–1129. 18313526 10.1016/j.ijrobp.2007.11.044

[10] Huang EH , Pollack A , Levy L , et al. Late rectal toxicity: dose‐volume effects of conformal radiotherapy for prostate cancer. Int J Radiat Oncol Biol Phys. 2002;54:1314–1321. 12459352 10.1016/s0360-3016(02)03742-2

[11] Mariados N , Sylvester J , Shah D , et al. Hydrogel spacer prospective multicenter randomized controlled pivotal trial: dosimetric and clinical effects of perirectal spacer application in men undergoing prostate image guided intensity modulated radiation therapy. Int J Radiat Oncol Biol Phys. 2015;92:971–977. 26054865 10.1016/j.ijrobp.2015.04.030

[12] Pinkawa M , Corral NE , Caffaro M , et al. Application of a spacer gel to optimize three‐dimensional conformal and intensity modulated radiotherapy for prostate cancer. Radiother Oncol. 2011;100:436–441. 21963289 10.1016/j.radonc.2011.09.005

[13] Saito M , Suzuki T , Sugama Y , et al. Comparison of rectal dose reduction by a hydrogel spacer among 3D conformal radiotherapy, volumetric‐modulated arc therapy, helical tomotherapy, CyberKnife and proton therapy. J Radiat Res. 2020;61:487–493. 32211861 10.1093/jrr/rraa013 PMC7299260

[14] Goldsmith C , Green MM , Middleton B , et al. Evaluation of CyberKnife(R) fiducial tracking limitations to assist targeting accuracy: a phantom study with fiducial displacement. Cureus. 2018;10:e3523. 30648058 10.7759/cureus.3523 PMC6318119

[15] Brand DH , Tree AC , Ostler P , et al. Intensity‐modulated fractionated radiotherapy versus stereotactic body radiotherapy for prostate cancer (PACE‐B): acute toxicity findings from an international, randomised, open‐label, phase 3, non‐inferiority trial. Lancet Oncol. 2019;20:1531–1543. 31540791 10.1016/S1470-2045(19)30569-8 PMC6838670

[16] Kataria T , Narang K , Bisht SS , et al. Analysis of intrafraction motion in CyberKnife‐based stereotaxy using mask based immobilization and 6D‐skull tracking. J Radiosurg SBRT. 2016;4:203. 29296445 PMC5658803

[17] Holmes OE , Gratton J , Szanto J , et al. Reducing errors in prostate tracking with an improved fiducial implantation protocol for CyberKnife based stereotactic body radiotherapy (SBRT). J Radiosurg SBRT. 2018;5:217. 29988326 PMC6018049

[18] Lei S , Piel N , Oermann EK , et al. Six‐dimensional correction of intra‐fractional prostate motion with cyberknife stereotactic body radiation therapy. Front Oncol. 2011;1:48. 22655248 10.3389/fonc.2011.00048 PMC3356099

[19] Xie Y , Djajaputra D , King CR , Hossain S , Ma L , Xing L . Intrafractional motion of the prostate during hypofractionated radiotherapy. Int J Radiat Oncol Biol Phys. 2008;72:236–246. 18722274 10.1016/j.ijrobp.2008.04.051 PMC2725181

[20] Sumila M , Mack A , Schneider U , Storelli F , Curschmann J , Gruber G . Long‐term intra‐fractional motion of the prostate using hydrogel spacer during Cyberknife® treatment for prostate cancer–a case report. Radiat Oncol. 2014;9:186. 25142237 10.1186/1748-717X-9-186 PMC4150956

[21] Langen KM , Jones DT . Organ motion and its management. Int J Radiat Oncol Biol Phys. 2001;50:265–278. 11316572 10.1016/s0360-3016(01)01453-5

[22] Onishi H , Kuriyama K , Komiyama T , et al. Large prostate motion produced by anal contraction. Radiother Oncol. 2012;104:390–394. 22673728 10.1016/j.radonc.2012.04.005

[23] Smeenk RJ , Louwe RJ , Langen KM , et al. An endorectal balloon reduces intrafraction prostate motion during radiotherapy. Int J Radiat Oncol Biol Phys. 2012;83:661–669. 22099035 10.1016/j.ijrobp.2011.07.028

[24] Picardi C , Rouzaud M , Kountouri M , et al. Impact of hydrogel spacer injections on interfraction prostate motion during prostate cancer radiotherapy. Acta Oncol. 2016;55:834–838. 26796870 10.3109/0284186X.2015.1128118

[25] Pinkawa M , Piroth MD , Holy R , et al. Spacer stability and prostate position variability during radiotherapy for prostate cancer applying a hydrogel to protect the rectal wall. Radiother Oncol. 2013;106:220–224. 23333015 10.1016/j.radonc.2012.11.010

[26] Juneja P , Kneebone A , Booth JT , et al. Prostate motion during radiotherapy of prostate cancer patients with and without application of a hydrogel spacer: a comparative study. Radiat Oncol. 2015;10:215. 26499473 10.1186/s13014-015-0526-1 PMC4619294

